# Single-gene association between *GATA-2* and autoimmune hepatitis: A novel genetic insight highlighting immunologic pathways to disease

**DOI:** 10.1016/j.jhep.2016.01.017

**Published:** 2016-05

**Authors:** Gwilym Webb, Yung-Yi Chen, Ka-Kit Li, Desley Neil, Ye Htun Oo, Alex Richter, Venetia Bigley, Matthew Collin, David H. Adams, Gideon M. Hirschfield

**Affiliations:** 1 Centre for Liver Research, University of Birmingham and NIHR Birmingham Liver Biomedical Research Unit, Birmingham, UK; 2 Department of Pathology, Queen Elizabeth Hospital, Birmingham, UK; 3 Department of Immunology, Queen Elizabeth Hospital, Birmingham, UK; 4 Institute of Cellular Medicine, Newcastle University, Newcastle, UK

**Keywords:** Regulatory T cell, Pathogenesis, Immune-mediated liver disease

## Abstract

Background & Aims

Autoimmune hepatitis (AIH), an immune-mediated liver disease, originates as a consequence of interacting genetic and environmental risk factors. Treatment remains non-specific and prone to side effects. Deficiencies in regulatory T cell (Treg) function are hypothesized to contribute to the pathogenesis of AIH.

Methods

We describe an adult patient who presented with AIH in the context of monocytopenia. The patient was characterized by *GATA2* gene sequencing, flow cytometry of peripheral blood for leucocyte subsets, ELISA for serum Flt-3 ligand, and immunohistochemistry of liver biopsy tissue.

Results

Sequencing confirmed a *GATA2* mutation. Peripheral Treg were absent in the context of a preserved total T cell count. Immunostaining for the Treg transcription factor FOXP3 was reduced in liver tissue as compared to a control AIH specimen. There were marked deficiencies in multiple antigen-presenting cell subsets and Flt-3 ligand was elevated. These findings are consistent with previous reports of *GATA2* dysfunction.

Conclusions

The association of a *GATA2* mutation with AIH is previously unrecognized. *GATA2* encodes a hematopoietic cell transcription factor, and mutations may manifest as monocytopenia, dendritic and B cell deficiencies, myelodysplasia, and immunodeficiency. Tregs may be depleted as in this case. Our findings provide support for the role of Tregs in AIH, complement reports of other deficiencies in T cell regulation causing AIH-like syndromes, and support the rationale of attempting to modulate the Treg axis for the therapeutic benefit of AIH patients.

## Introduction

Autoimmune hepatitis (AIH) is an immune-mediated liver disease with environmental and genetic risk factors. Deficits in immunoregulation, most notably regulatory T cell (Treg) function, are associated with etiopathogenesis [1].

HLA associations with AIH represent the strongest genetic risk factors, implying specific immune presentation of triggering antigens [2]. Rare Mendelian genetic variation is additionally mechanistically informative, with strong association between AIH and recessive mutations in the autoimmune regulator gene, *AIRE* [3]. Such mutations prevent the thymic medulla presenting tissue-restricted antigens to developing T cells, impairing both negative selection of autoreactive cells and generation of self-specific Tregs. Furthermore, murine immune-mediated hepatitis can be generated by medullary thymic epithelial cell depletion through deletion of *TRAF6* and Treg deficiency causes peri-portal inflammation [4], [5].

We report and characterize AIH associated with a mutation in *GATA2*, a novel observation with mechanistic and therapeutic insights.

## Patient

Our patient, of European Caucasian ancestry and without family history of note, presented at age 27, but gave a history of lymphedema, possible porphyria cutanea tarda, and intermittent superficial skin infections as a teenager. Aged 21 she developed trilineage cytopenias including monocytopenia initially diagnosed as myelodysplastic syndrome (MDS). Bone marrow aspiration revealed absolute loss of multi-lymphoid and granulocyte-macrophage progenitors in a hypocellular marrow. She developed an erythrocyte transfusion requirement and her infections became more frequent.

**Fig. 1:**
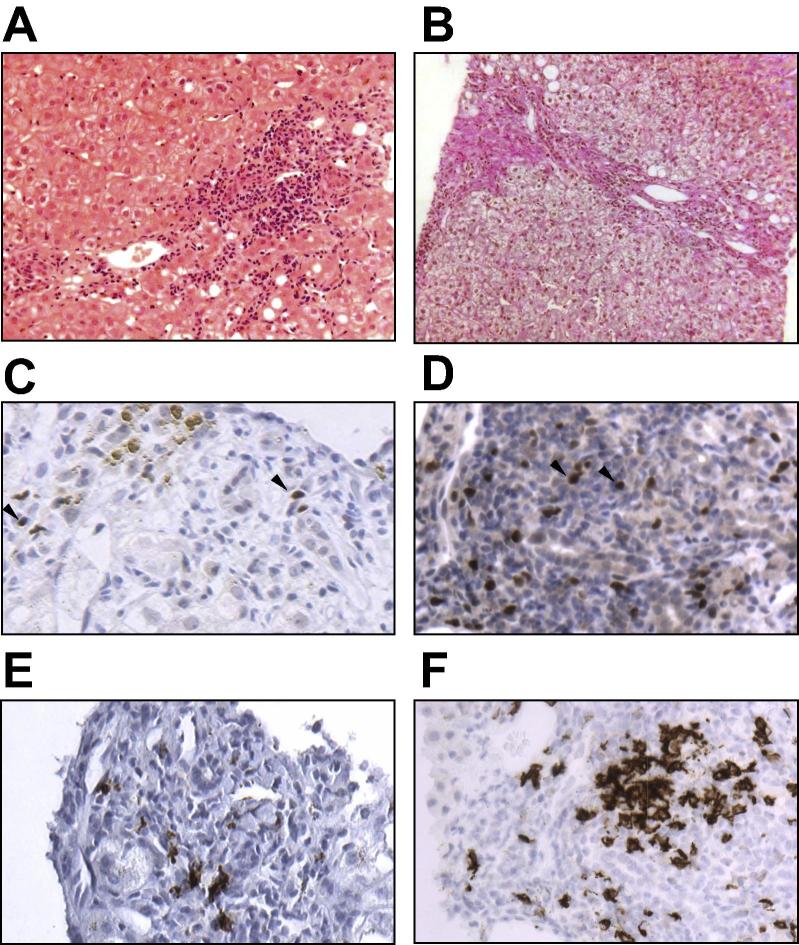
**Liver histopathology.** (A) Transjugular liver biopsy sample demonstrating dense lymphocytic infiltrate with interface hepatitis consistent with AIH (haematoxylin and eosin; 20×). (B) Fibrosis bridging between portal areas (Van Gieson; 20×). (C and D) Cells positive for the Treg transcription factor FOXP3 were scant in the inflammatory infiltrate of patient liver (C), but more frequent in a control AIH sample (D) (both 32×; example positive staining denoted by arrowheads). (E and F) CD20-positive B cells were present in patient liver biopsy specimen (E) in contrast to peripheral blood but at a reduced frequency to a control AIH sample (F; 32×). (This figure appears in colour on the web.)

Aged 27, she developed elevated liver biochemistry consistent with hepatitis, which resolved spontaneously. Aged 28, whilst free of medical immunosuppression, she developed hepatitis with ascites. Ultrasound and magnetic resonance cholangiopancreatography showed hepatosplenomegaly and showed no evidence of biliary disease. Total IgG was elevated peaking at 41.05 g/L, polyclonal and predominantly IgG1. Anti-nuclear antibodies were positive at 1:100 in a speckled pattern; other autoantibodies including anti-mitochondrial antibody were negative (Supplementary Tables 1 and 2). Liver biopsy revealed plasma cells and interface hepatitis consistent with AIH (Fig. 1). There was mild macrovesicular steatosis, moderate iron deposition consistent with previous repeated transfusions and moderate fibrosis; staining suggestive of alternate etiologies was negative, including specific staining for Epstein-Barr virus (EBV; Supplementary materials). An EBV viral load of 10^4^–10^5^ copies/ml was present throughout; no other viral factors were identified including negative PCR for hepatitis B, C, E and cytomegalovirus. Human leukocyte antigen (HLA) genotyping revealed non-AIH risk alleles: *HLA-DPB1∗03:01* and *HLA-DPB1∗10:01* (Supplementary Table 3). Functional antibody testing confirmed preserved ability to generate antigen-specific responses (Supplementary Table 4).

Corticosteroids were commenced as prednisolone 40 mg/day, and her ascites and liver biochemistry tests resolved. Shortly afterwards, she developed JC/polyoma virus-positive progressive multifocal leukoencephalopathy (PML). Corticosteroids were discontinued, PML treatment commenced, and she regained the ability to walk. On discontinuing corticosteroids, her liver biochemistry again deteriorated. Over subsequent years she received variable corticosteroid-tacrolimus immunosuppression without recurrence of PML but with varying elevations in transaminases.

Repeat liver biopsy at the age of 32 showed similar features with progressing fibrosis. She later developed human papilloma virus-associated vulval carcinoma, which was treated with radiotherapy. At this point investigations were initiated for suspected *GATA2* mutation.

After investigations confirmed *GATA2* mutation, hematopoietic stem cell transplantation was performed. The allograft was unsuccessful and the patient ultimately died from complications of vulval carcinoma.

## Results

**Fig. 2:**
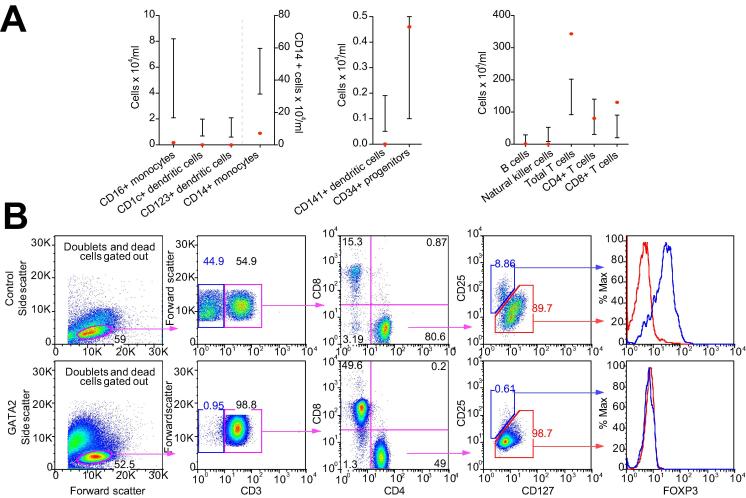
**Immunophenotyping by flow cytometry.** (A) Flow cytometric analysis of peripheral blood demonstrated antigen-presenting cell deficiencies and leukocytopenias in multiple subsets with preserved total T cells. (B) Analysis of T cell populations revealed minimal CD25^low^CD127^hi^ cells and no evidence of expression of the transcription factor FOXP3 in a patient sample. This confirmed a marked deficiency in Treg. Approximately 8% of peripheral CD4+ T cells were CD25^low^CD127^hi^FOXP3^+^Treg in a control sample. The absence of CD3-negative cells (blue gate, second panel) highlights B- and NK-cell deficiency. CD4:CD8 ratio was altered from the CD4-predominance seen in normality to equality, as reported in GATA2 dysfunction. (This figure appears in colour on the web.)

DNA sequencing revealed a coding 1081C>T R361C abnormality in exon 7 of *GATA2*. Serum Fms-like tyrosine kinase 3 ligand (Flt-3L) was markedly elevated at 1267.2 pg/ml (normal 48.3–173.8 pg/ml); sequencing for Flt-3 receptor mutations was negative. Flow cytometry demonstrated marked reductions in numbers of dendritic cells, monocytes, B and natural killer (NK) cells in keeping with ‘DCML deficiency’ [6] (Table 1). There was a maintained T cell population but near absence of FOXP3+ Treg (Fig. 2; Supplementary Fig. 1). Sparse FOXP3+ cells were seen in the hepatic inflammatory infiltrate and fewer than when compared to control AIH, or prior reports (Fig. 1) [7]. *In situ* hybridization staining was negative for EBV (Supplementary Fig. 2).

**Table 1:** **Leucocyte subtypes.**

The calculated AIH score according to the International autoimmune hepatitis group revised diagnostic scoring system was 21 with a score >17 suggesting definite AIH (Table 2).

**Table 2:** **International autoimmune hepatitis group revised diagnostic scoring system.**

∗HLA type = HLA-DPB1∗03:01, DPB1∗10:01.

## Discussion

Specific therapy in AIH is limited by our understanding of disease etiopathogenesis. Here we demonstrate the association of a missense mutation in *GATA2*, a hematopoietic transcription factor, with classical AIH and systemic Treg deficiency. This new molecular insight in AIH supports the relevance of developing novel therapies focused on reconstituting regulatory balance.

GATA2 dysfunction may present in the first two decades with lymphedema, deafness and myelodysplasia (Emberger syndrome), or acute myeloid leukemia without preceding immunodeficiency [8]. Presentation may also be with non-tuberculous mycobacterial infections and monocytopenia, DCML deficiency or familial MDS/acute myeloid leukemia [9], [10]. Related carriers may remain healthy for decades. The cause of the variable age of onset may be unidentified environmental exposures or additional genetic risk, for example *AXL1* mutation [5]. Most individuals with GATA2 dysfunction develop MDS; elevated serum Flt-3 ligand is almost universal [11]. Monocytopenia is a vital clue to this rare diagnosis; chronic neutropenia and NK deficiency may also be suggestive [9]. This is the first characterization of associated AIH, but autoimmune phenomena including arthritis and hypothyroidism are recognized [11].

Several features support the diagnosis of AIH: plasma cells and interface hepatitis on liver biopsy, steroid responsiveness with relapse on withdrawal, the presence of anti-nuclear antibody and elevated serum IgG. IgG is usually normal in GATA2 dysfunction, despite the near absence of peripheral B cells [5]. In our case, B cells were present in the hepatic inflammatory infiltrate but not peripherally, suggesting local induction. The differential diagnosis of EBV-driven hepatitis was made unlikely by steroid responsiveness, stable viremia and negative *in situ* hybridization assay. We note that EBV viremia was reported amongst 11% of one cohort of patients with monoMAC but hepatitis was not reported [8].

The increase in absolute and relative numbers of CD8 T cells in the peripheral blood of this patient is of interest. CD8 cells have been implicated in the pathogenesis of AIH: they are present in the inflammatory infiltrate, cells isolated from those with active disease may be resistant to regulation be more resistant than usual to regulation by Treg [1]. In addition, several animal models of AIH demonstrate increases in both peripheral and hepatic CD8+ T cell numbers [1]. Serum soluble CD8, which is increased in CD8 T cell activation, is also reported to be elevated in AIH. Although it is tempting to link the elevation in CD8+ T cells to AIH, it is notable that accumulation of CD8+ T cells is a feature of progressing GATA2 dysfunction without AIH [11].

Of particular interest in this patient is the Treg deficiency despite preserved numbers of total CD4+ and CD8+ T cells. Tregs may be depleted in association with dendritic cell deficiency, including in GATA2 dysfunction [12], [13]. We speculate that this deficiency may have contributed to a loss of immunological tolerance. Sparse FOXP3 staining on liver biopsy may reflect activated T cells transiently expressing FOXP3 or alternatively induced/trans-differentiated Treg in an inflammatory environment [14]. Reduced CD127 expression on peripheral T cells was consistent with impaired memory cell induction as reported in GATA2 dysfunction [11]. This may have contributed to a susceptibility to viral infections.

The association of a functional mutation in *GATA2* with AIH and Treg deficiency informs efforts to further examine regulatory mechanisms in AIH to better understand pathogenesis and guide rational therapy.

## Conflict of interest

The authors who have taken part in this study declared that they do not have anything to disclose regarding funding or conflict of interest with respect to this manuscript.

## Authors’ contributions

GW drafted the manuscript and analyzed patient samples; YC, KL and DN assisted with sample analysis; YO, AR, VB, MC, DA and GMH were involved with patient care and VB also analyzed patient samples; GMH conceived the study, finalized the manuscript and acts as guarantor. All authors approved submission.
