# Supplementary material for: Single-gene association between GATA-2 and autoimmune hepatitis: A novel genetic insight highlighting immunologic pathways to disease
Source: J Hepatol. 2016 May;64(5):1190–3. doi: 10.1016/j.jhep.2016.01.017 (PMC4824751; doi:10.1016/j.jhep.2016.01.017)
Supplement: Supplementary data [file mmc1.pdf]

Single-gene association between *GATA-2* and autoimmune hepatitis: A novel genetic insight highlighting immunologic pathways to disease

Gwilym Webb, Yung-Yi Chen, Ka-Kit Li, Desley Neil, Ye Htun Oo, Alex Richter, Venetia Bigley, Matthew Collin, David H Adams, Gideon M Hirschfield

Table of contents

Supplementary material……………………………………………………………….2

Supplementary Fig. 1…………………………………………………………………5

Supplementary Fig. 2…………………………………………………………………6

Supplementary Table 1……………………………………………………………….7

Supplementary Table 2……………………………………………………………….9

Supplementary Table 3……………………………………………………………...12

Supplementary Table 4……………………………………………………………...14

Supplementary Table 5……………………………………………………………...16

Supplemental materials

Clinical laboratory indices and ANAs

Routine laboratory data were gathered from hospital laboratory analyses; ANAs were assessed by indirect immunofluorescence staining of Hep-2 slides (Inova diagnostics, San Diego, USA) visualized with FITC Mouse Anti-Human IgG (Biosciences, Oxford, UK).

Histology and Immunohistochemistry

Liver biopsy specimens were stored in 10% formaldehyde-saline solution until embedded in paraffin and then cut into 4µM sections. Sections were subsequently dewaxed using Clearene® (Leica Biosystems, Newcastle Upon Tyne, UK), rehydrated using graded alcohols and water and then either stained with haematoxylin and eosin or Van Gieson stain using standard techniques or for specific antigens as below.

EBV-staining and in situ hybridization was performed using peptide nucleic acid kit K5201 and EBV virus probe Y5200 (both Dako UK Ltd, Cambridgeshire, UK) according to the manufacturer's instructions.

For FoxP3 staining, tissues non-specific staining was blocked with methanol-hydrogen peroxide solution; antigen retrieval was performed by emersion in a high pH EDTA solution (Vector Laboratories Ltd, Peterborough, UK) and heating with a microwave; further non-specific staining was reduced by incubation with Casein solution (Vector); FoxP3 was stained with rat anti-human FoxP3 Biotin antibody (Clone PCH101, eBioscience, San Diego, CA, USA). B cells were stained with mouse anti-human CD20 antibody (Clone L26,

Dako). Staining was visualised with anti-mouse or anti-rat peroxidase and 3, 3'-diaminobenzidine substrate (Vector). Sections were counter-stained with Mayer's haematoxylin.

Flow cytometry

Peripheral blood mononuclear cells were isolated from whole, EDTA-anticoagulated blood by density gradient centrifugation using Lympholyte®-H (Cedarlane, Burlington, Canada). They were stored until analysed in media supplemented with fetal calf serum and 10% DMSO in liquid nitrogen until analysis.

Control blood was obtained from an otherwise healthy patient with genetic haemochromatosis undergoing routine therapeutic venesection.

For profiling regulatory T-cells, the following fluorophore-conjugated antibodies were used with appropriate isotype controls from BD Biosciences: CD3-allophycocyanin (SK7), CD4-v500 (RPA-T4), CD8-Phycoerythein-Cyanine7 (RPA-T8), CD25-Brilliant Violet 421 (M-A251), CD127-fluorescein isothiocyanate(HIL-7R-M21). Zombie-NIR™ was used to exclude dead cells from analysis (Biolegend, London, UK).

Intracellular staining for FOXP3 was performed using a transcription factor buffer staining kit (eBioscience) and FOXP3-phycoerythrin antibody (BD Biosciences, Oxford, UK). Samples were analysed on a CyAn ADP analyser (Beckman Coulter, Brea, CA, USA) and analysed using FlowJo 8.7 software (TreeStar, Ashland, OR, USA).

For obtaining peripheral cell counts, the following fluorophore-conjugated antibodies were used with appropriate isotype controls (all BD unless otherwise stated): CD1a FITC (NA1/34; Dako); CD1a APC (HI 149); CD3 FITC, PE & APC (UCHT1) PerCPCy5.5 (SK7); CD4 FITC (SK3); CD8 APC-Cy7 (SK1); CD10 APC (HI10a); CD11c APC & V450 (B-ly6); CD14 PE, PE-Cy7 (M5E2) & Q-DOT605 (TuK4; Invitrogen); CD16 FITC, PE & PE-Cy7 (3G8); CD19 FITC, PE & PE-Cy7 (SJ25C1); CD25 PE & PE-Cy7 (2A3); CD34 APC (8G12) and APC-Cy7 (581; BioLegend); CD38 PE-Cy7 (HB7); CD45 APC-Cy7 & V450 (2D1); CD45RA FITC (L48) & PE (HI100); CD56 FITC & PE (NCAM16.2); CD90 Q-DOT605 (5E10; eBioscience); CD123 PE (9F5); CD135 PE (4G8); HLA-DR PerCP-Cy5.5 (L243); and Langerin PE (DCGM4; Beckman Coulter). Flow cytometry was performed on an LSR Fortessa X20 (BD Biosciences).

Sequencing of GATA-2

Peripheral blood was used as the source of DNA. Genomic DNA was extracted using the QIAamp DNA Mini Kit (QIAGEN), while polymerase chain reaction (PCR) amplification and Sanger sequencing was performed using primers and conditions described previously.[11]

Measurement of Flt3 ligand

Serum Flt3 ligand was measured by ELISA kits according to the manufacturer's instructions (Quantikine Human Flt3/Flk-2 Ligand Immunoassay, R&D Systems, Abingdon, UK).

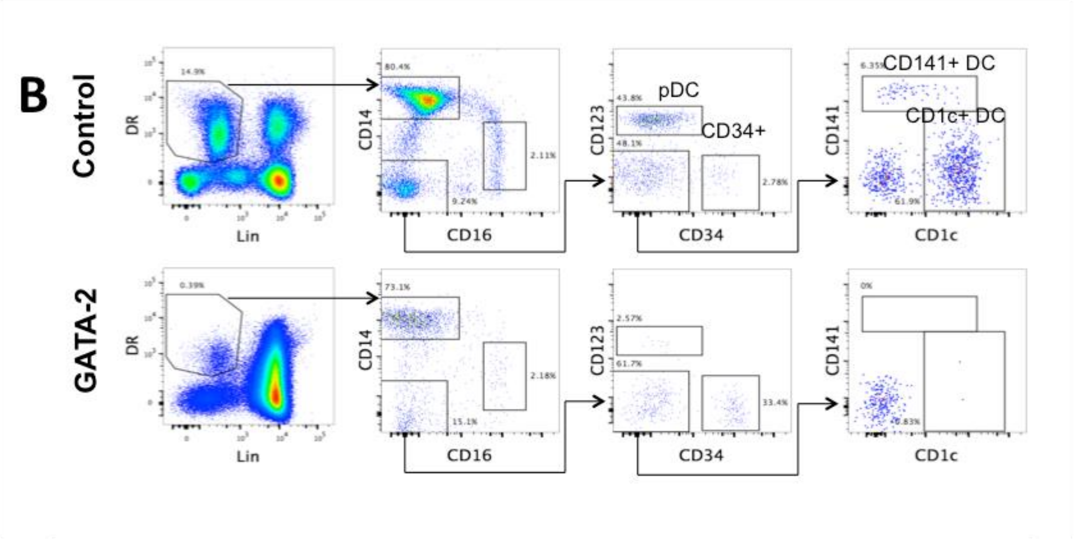

Supplementary Fig. 1. Gating strategy for dendritic cells and monocytes

Flow cytometric gating strategy for monocytes and dendritic cells demonstrating near absence of both subsets in a sample of leucocytes isolated from patient peripheral blood as compared with a control sample.

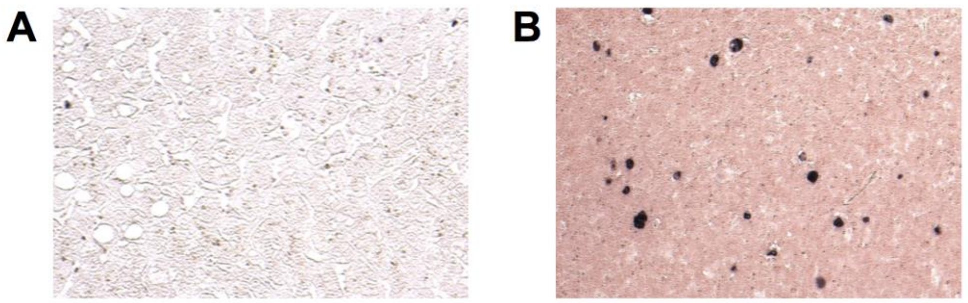

Supplementary Fig. 2. Negative EBV in situ hybridization on liver biopsy sample

EBV *in situ* hybridization showed no significant staining in patient sample (A) compared with control positive EBV hepatitis specimen suggesting that hepatocyte infection associated with low-level EBV viremia was not inducing a specific hepatic immune response (B)(both ×10).

Supplementary Table 1. Baseline laboratory variables

Variable	Value	Normal range	Units
Bilirubin	16	< 21	µmol/L
Alanine aminotransferase	110	< 41	iu/L
Alkaline phosphatase	315	20 – 130	iu/L
Albumin	32	35 – 50	g/L
Creatinine	50	44 – 133	µmol/L
C-reactive protein	11	< 5	mg/L
International normalised ratio	1.2	0.8 – 1.2	
Hemoglobin	98	115 – 160	g/L
Platelets	40	150 – 450	$\times 10^{12}$/L
White cell count	4.4	4 – 11	$\times 10^{9}$/L
Neutrophils	2.99	1.7 – 7.5	$\times 10^{9}$/L
Lymphocytes	1.27	1 – 4.5	$\times 10^{9}$/L
Monocytes	0.07	0.2 – 0.8	$\times 10^{9}$/L

Eosinophils	0.01	< 0.5	$\times 10^9$/L
Immunoglobulin G	36.9	5.3 – 16.5	g/L
Immunoglobulin A	1.22	0.8 – 4	g/L
Immunoglobulin M	1.9	0.5 – 2.5	g/L

Baseline laboratory values: values closest to date of diagnosis presented.

Supplementary Table 2. Autoantibody testing results

Autoantibody tested	Method	Result
Anti-nuclear antibody	ELISA and Indirect immunofluorescence	Positive at 1:100; speckled pattern on immunofluorescence.
Anti-mitochondrial antibody	ELISA / Indirect immunofluorescence	Negative
LC-1*	Immunoblot	Negative
LKM-1	Immunoblot	Negative

SLA/LP*	Immunoblot	Negative
gp210*	Immunoblot	Negative
Sp100*	Immunoblot	Negative
f-Actin*	Immunoblot	Negative
Gastric parietal cell	Immunoblot and indirect immunofluorescence	Negative
Smooth muscle	Immunoblot and indirect immunofluorescence	Negative

Intrinsic factor	Immunoblot and indirect immunofluorescence	Negative

Tests marked with a star (*) were performed prior to the introduction of immunosuppression; those without were performed after the introduction of corticosteroids. LC-1 = liver cytosol antibody type 1; LKM-1 = liver kidney microsomal antibody 1; SLA/LP = soluble liver antigen / liver pancreas; gp210 = nuclear pore glycoprotein 210; F-actin = filamentous actin; ELISA = enzyme-linked immunoabsorbance assay

Supplementary Table 3. HLA genotyping

Test results:

HLA-DPB1*03:01, DPB1*10:01

Definitive results:

HLA-A*02:01/01L/04/07/09/12/15N/17/18/20/24/25/27/29-31/33/36/42/43N/53N/59/60/64/66/67/73-76/82N/83N/85N/88N/89/93/94N/95-97/101/105/107/109-111/114/119-121/123/132-134/138-141/145/147/150/151/157/160-165/167/168/173/175-77/181/183/187-189/192-194/196/197/199-201/203-208/210-216/218-221/222N/223N/224/225N/227N/228/231/235/236/238/240-242/245/246/250N/251/252/254-257/263/265/266/268-270/272-277/279/282/284N/285/287-289/291/292/293Q/294/296/299/301N;

A*24:02/03/09N/11N/13-15/17/20/21/25/26/33/35/36N/37-39/40N/41/45N/47/48N/49/51/53/58/59/63/64/68-70/72-74/76/78-81/83N/84N/85/86N/88/90N/93/95/96/98/99/101-106/110/111/113-118/120-123/125-128/132N/135-137/139-144/146-154/155N/157/158N/161/162/162/163N/165/166/169-171;

B*15:01/01N/26N/27/33-35/60/71/78/79N/82/92/96/102/104/110/118/122/129/140/141/146/147/154/159/160/165-167/169/171/172/174/175/178/181N/182N/184/187/190N/192/201/203/205/206/211;

B*44:03/13/26/32/36/38/39/65/69/85/94/96/98/103/108N/110/114/115/122/125;

C*03:03/11/20N/22Q/30/43/50/52/53/56/62/66/68/69/75/79/81/83/85/88/102, C*16:01

HLA-DRB1*04:04, DRB1*11; DRB3*02:02/08; DRB4*01:01-05; DQB1*03:01/27-29/35/42/47, DQB1*03:02P; DPB1*03:01, DPB*10:01

Performed by PCR-SSOP

Supplementary Table 4. Specific antibody testing

Antibody	Result	Positive result
Haemophilus B abs	0.93 ug/ml	>1.00
Tetanus abs	0.33 IU/ml	>0.10
Diphtheria abs IgG	0.02 IU/ml	>0.10
Meningitis serotype A IgG	9.30 ug/ml	>2.00
Meningitis serotype C IgG	1.74 ug/ml	>2.00
Meningitis serotype W IgG	0.36 ug/ml	>2.00
Meningitis serotype Y IgG	0.37 ug/ml	>2.00
Pneumococcus serotype 1 IgG	0.31 ug/ml	>0.35
Pneumococcus serotype 14 IgG	0.88 ug/ml	>0.35
Pneumococcus serotype 18c IgG	0.05 ug/ml	>0.35
Pneumococcus serotype 19a IgG	0.32 ug/ml	>0.35
Pneumococcus serotype 19f IgG	0.24 ug/ml	>0.35
Pneumococcus serotype 23f IgG	0.30 ug/ml	>0.35
Pneumococcus serotype 3 IgG	0.08 ug/ml	>0.35
Pneumococcus serotype 4 IgG	0.04 ug/ml	>0.35

Pneumococcus serotype 5 IgG	0.06 ug/ml	>0.35
Pneumococcus serotype 6b IgG	0.03 ug/ml	>0.35
Pneumococcus serotype 7f IgG	0.50 ug/ml	>0.35
Pneumococcus serotype 9v IgG	0.05 ug/ml	>0.35

Supplementary Table 5. Histological scoring

Steatosis was graded as mild (according to the semi-quantitative method of Neuschwander-Tetri and Caldwell).
The non-alcoholic fatty liver disease (NAFLD) score was 4 (Kleiner), which is not diagnostic for non-alcoholic steatohepatitis.
Fibrosis was graded as mild-moderate on the IASL system and Stage 2-3 by the Batts-Ludwig system because of the presence of several bands of bridging fibrosis.
